# Association between albumin and short-term outcomes of unplanned early readmission emergency department patients: A retrospective cohort study

**DOI:** 10.1371/journal.pone.0327501

**Published:** 2025-07-24

**Authors:** Liang Zhou, Xiaona Cai, Kunpeng Hu, Shuo Tang, Dandan Gao, Xiaojuan Zhang, Yin Wu, Li He, Bangfu Zhou, Ruoyu Wu, Zhigang Zhang

**Affiliations:** 1 State Key Laboratory of Trauma and Chemical Poisoning, Department of Field Medical Equipment, Daping Hospital, Army Medical University, Chongqing, China; 2 Department of Clinical Laboratory Medicine, Daping Hospital, Army Medical University, Chongqing, China; Pelita Harapan University Faculty of Medicine: Universitas Pelita Harapan Fakultas Kedokteran, INDONESIA

## Abstract

**Objective:**

This study was a post hoc analysis of data from a retrospective cohort population. The purpose of this study was to analyze the predictive value of albumin for the short-term survival of patients with unplanned early readmission to the emergency department (ED).

**Methods:**

A total of 6,249 emergency patients were enrolled in this study, including 5,368 patients (85.49%) who had been admitted for the first time and 881 patients (14.51%) with unplanned early readmission to the ED; the data were from the clinical cohort data publicly released on the Dryad website. The patients were classified on the basis of the tertile of their albumin values. Kaplan‒Meier survival curve analysis, the log-rank test and a Cox proportional hazards model were used to analyze the associations between albumin levels and 30-day mortality risk in emergency patients. The dose‒response relationship between albumin and outcome was fitted by a restricted cubic spline (RCS). Sensitivity analysis was performed on the results of the difference test by creating a multivariate Cox regression model with the forward likelihood ratio (LR) method.

**Results:**

The demographic characteristics and clinical indicators of patients in the ED differed according to the number of admissions. The median albumin level of patients admitted to the hospital for the first time was greater than that of patients who were readmitted (Z = −9.642, *P* < 0.001). The duration of patient hospitalization was used as the follow-up time. The median length of hospitalization was 2 days (range: 1–123 days); 328 patients died within 30 days, and the all-cause mortality rate of patients with unplanned early readmission was higher than that of first-time hospitalization patients (4.49% vs. 9.86%, $\chi^{2}$ =44.136, *P* < 0.001). Kaplan–Meier survival curve analysis revealed that albumin levels were associated with 30-day mortality in all patients, first-time admitted patients, and patients with unplanned early readmission (log-rank test, *P* < 0.001). The multivariate Cox regression model revealed that albumin was a significant factor only in all patients combined (HR 0.93, 95% CI 0.91–0.95, *P* < 0.001) and in first-time admitted patients (HR 0.92, 95% CI 0.89–0.94, *P* < 0.001) but not in patients with unplanned early readmission (HR 0.97, 95% CI 0.93–1.01, *P* = 0.121). The same result was obtained with a Cox regression model in which albumin was included as a categorical variable. Sensitivity analysis revealed that the results of multivariate Cox regression analysis were reliable.

**Conclusion:**

There was a specific correlation between albumin and the short-term outcome of emergency patients. Albumin levels are associated with short-term outcomes in patients admitted to the hospital for the first time, but its association with the outcomes of patients with unplanned early readmission to the ED is likely trivial.

## Introduction

Early and accurate assessment of the severity of emergency department (ED) patients’ conditions not only reduces adverse clinical outcomes but also optimizes resource allocation, thereby improving both healthcare service quality and patient satisfaction [1–3]. While EDs traditionally rely on vital signs for triage decisions, rapid biomarker detection technologies now increase the accuracy of identifying critically ill patients [4,5]. Selecting validated biomarkers improves risk stratification efficiency, directly optimizing both clinical care quality and resource utilization [6–8].

Albumin is the most abundant protein in human serum. Its half-life is approximately 21 days, and it is susceptible to reduced synthesis and accelerated metabolism as a result of disease, which in turn leads to a rapid decrease in its serum concentration [9]. Therefore, albumin is often used as a biomarker to assess human health status. Studies have shown that hypoalbuminemia in ED patients may be associated with disease severity, which is attributed to suppressed albumin synthesis and accelerated consumption caused by abnormal liver metabolism or inflammatory responses [10,11].

Unplanned early readmission (within 30 days postdischarge) among ED patients is strongly associated with unresolved clinical deterioration or iatrogenic complications [12,13]. Previous studies revealed that for most diseases, the outcomes of patients readmitted to the ED are poor [14,15]. Early identification of these patients’ conditions enables targeted interventions to mitigate adverse clinical outcomes. Indeed, hypoproteinemia has been associated with an increased risk of readmission for many patients [16–20]. Studies have reported that patients with kidney disease or malignant tumors have a high risk of readmission in the short term after surgery [16,21] and are likely to experience malnutrition, inflammation, metabolic disorders and other conditions, which results in a decrease in albumin [22–25]. However, current evidence suggests that hypoalbuminemia is more likely to be a secondary pathological change rather than an initiating factor in disease progression [26]. Evidence-based medical data show that increasing the plasma albumin concentration through exogenous supplementation has significant limitations in improving disease prognosis [10,27,28]. This pathological and physiological characteristic limits the utility of albumin levels as an independent diagnostic indicator, making it difficult to accurately assess disease activity, and albumin levels lack clinical significance in guiding specific treatment decisions.

Although albumin is used as an important indicator in laboratory tests for ED patients, the efficacy of albumin in predicting short-term outcomes among patients with unplanned early readmission to the ED, who have low albumin levels, remains to be verified. Currently, few studies have investigated the correlation between albumin and survival in patients with unplanned early readmission to the ED, resulting in ambiguity and controversy regarding this relationship. Therefore, the aim of this study was to perform a post hoc analysis of data from a retrospective cohort to analyze the predictive value of albumin for the short-term outcomes of patients with unplanned early readmission to the ED.

## Methods

### Data sources and study population

This study was a post hoc analysis of clinical cohort data publicly available on the Dryad website (which is an open data publishing platform and a community committed to the open availability and routine reuse of all research data), which has strict quality review and program management processes to ensure the authenticity and availability of the published data. The data of the patients included in this study have been published previously, and no personal privacy information is included. The data acquisition, analysis, and reporting processes were performed with ethical permission from the Danish National Committee on Biomedical Research Ethics (J.nr. HA-2009–006) and the Danish Data Protection Agency (J.nr. HIH 2009–2 Akutdatabasen and J. nr. 2007–58-0015); for minors (<18 years), informed consent for participation in the study was signed by a guardian or parent [29]. The complete dataset is publicly accessible online (https://datadryad.org/stash/dataset/doi:10.5061/dryad.m2bq5).

Patient information was obtained from the ED of Nordsjælland University Hospital. The patients were registered in the hospital system between September 22, 2009, and February 28, 2010, and included 6,249 emergency patients who were referred to or sought medical care, which included 5,368 first-admitted patients and 881 patients with unplanned early readmission (<30 days postdischarge). The first-admission ED patients included 2,786 males and 2,582 females (aged 16–108 years), whereas the patients with early unplanned readmission to the ED included 450 males and 431 females (aged 16–99 years). The reasons for admission included severe trauma, ST-segment elevation myocardial infarction, or stroke and other life-threatening acute symptoms. The admission time was limited to within 3 hours of the onset of symptoms. Other patient factors (sex, race, severity of condition, etc.) were not considered in the eligibility criteria.

### Demographic and clinical data

Five items were included: I. demographic characteristics, including age and sex; II. physical examination results, including systolic blood pressure, heart rate, respiratory rate, oxygen saturation, and Glasgow Coma Scale score; III. laboratory examination results, including albumin, C-reactive protein, sodium, potassium, hemoglobin, creatinine, white blood cell count, platelet count, and lactate dehydrogenase; IV. triage information, coded as red (resuscitation), orange (emergent), yellow (urgent) or green (nonurgent) [29]; and V. hospital admission information, including readmission status within 30 days and length of hospitalization.

### Blood sample measurement

Within 1 hour after patient admission, a phlebotomist or medical student receiving training in blood collection drew samples of venous blood. A Dimension Vista®1500 (Siemens Medical Solutions Diagnostics, Erlangen, Germany) was used to measure the levels of albumin, C-reactive protein (CRP), hemoglobin, sodium, potassium, creatinine and lactate dehydrogenase [30]. A Sysmex Xie-5000 (Sysmex Corporation, Kobe City, Japan) was used to obtain the white blood cell count.

### Outcomes

The primary outcome was death within 30 days of patient admission to the hospital.

### Statistical analysis

For demographic and clinical data, continuous variables are expressed as medians and interquartile ranges (IQRs), and categorical variables are expressed as frequencies and percentages. Intergroup differences in continuous variables were analyzed with the Mann‒Whitney U test or the Kruskal‒Wallis H test, and intergroup differences in categorical variables were analyzed with the $\chi^{2}$ test at a significance level of α = 0.05 (the results of the normality analysis of continuous variables are shown in S1 Table in S1 File). Missing data were addressed with multiple imputation. The emergency patients were divided into “first admission” and “readmission” groups according to the number of hospitalizations, and the differences in albumin and 30-day mortality between the two groups were evaluated. Kaplan‒Meier survival curve analysis and the log-rank test were used to evaluate the overall survival of the patients. The correlation between the albumin and 30-day mortality risk under multivariable conditions was evaluated via a Cox proportional hazards model. Variables with *P* < 0.05 in univariable analyses were entered into multivariate Cox regression analysis and used as adjusted covariates during model fitting. The endpoint evaluation indicators were the hazard ratio (HR) and 95% confidence interval (CI). Considering possible collinearities among the covariates, a multivariate Cox regression model with the forward likelihood ratio (LR) method was used to perform sensitivity analysis on the results of the difference test. Additionally, the results of differential analysis for albumin in different groups (first, divided by albumin tertiles; second, divided by an albumin threshold value of <35.0 g/L vs ≥ 35.0 g/L) were used as sensitivity analysis methods. The dose‒response relationship between albumin and 30-day mortality was fitted by a restricted cubic spline (RCS). To ensure the best fitting result of the RCS, the number of nodes of the RCS. was set to 4, and a two-tailed *P* < 0.05 was considered statistically significant. Data analysis was performed with IBM SPSS Statistics version 25 (IBM, Armonk, New York) and R (version 4.4.0) software (R Foundation for Statistical Computing, Vienna, Austria).

## Results

### Baseline clinical characteristics

The included sample size was 6,249 patients, including 5,368 patients (85.49%) who were admitted to the ED for the first time and 881 patients (14.51%) with unplanned early readmission to the ED. We divided the patients into groups according to the tertiles of the albumin value. Differences in the baseline data between the tertile groups are summarized in Tables 1 and 2. The results revealed differences in sex and triage information only among ED patients admitted to the hospital for the first time (*P* < 0.001). There was no difference in potassium between first-time admitted patients and patients with unplanned early readmission (*P* > 0.05); differences in the other indicators among the tertile groups were present in both populations (*P* < 0.05).

**Table 1:** Baseline demographic and clinical characteristics of first-admission patients.

	All (n = 5,368)	Tertile 1 (n = 1,798)	Tertile 2 (n = 1,775)	Tertile 3 (n = 1,795)
Albumin, g/L	42.2 (38.9-44.8)	<40.1	40.1-43.9	>43.9
Age, year	62 (44-75)	70 (58-81)	62 (45-74) †	51 (36-65) †‡
Sex				
Male, n (%)	2786 (51.9)	957 (17.8)	969 (18.1)	860 (16.0) †
Female, n (%)	2582 (48.1)	841 (15.7)	806 (15.0)	935 (17.4)
Triage				
Green, n (%)	1729 (32.2)	502 (9.4)	570 (10.6)	657 (12.2) †
Yellow, n (%)	2045 (38.1)	732 (13.6)	659 (12.3)	654 (12.2)
Orange, n (%)	1361 (25.4)	464 (8.6)	482 (9.0)	415 (7.7)
Red, n (%)	233 (4.3)	100 (1.9)	64 (1.2)	69 (1.3)
C-reactive protein, mg/L	0.6 (0.2-2.9)	2.5 (0.6-9.9)	0.5 (0.2-2.1) †	0.27 (0.1-0.8) †‡
Potassium, mmol/L	4.1 (3.8-4.3)	4.0 (3.8-4.4)	4.1 (3.8-4.3)	4.1 (3.8-4.3)
Sodium, mmol/L	137.6 (135.0-139.5)	136.4 (133.1-138.8)	137.8 (135.7-139.5) †	138.2 (136.6-140.0) †‡
Hemoglobin, mmol/L	8.4 (7.6-9.1)	7.6 (6.8-8.4)	8.4 (7.8-9.0) †	8.9 (8.4-9.5) †‡
Creatinine, μmol/L	71.1 (59.0-86.1)	74.0 (59.3-100.7)	69.8 (58.2-84.5)	69.9 (59.1-81.5) †‡
Blood leucocyte count, 109/L	8.7 (6.8-11.5)	9.1 (7.1-12.4)	8.5 (6.7-11.0)	8.5 (6.6-11.2) †‡
Lactate dehydrogenase, U/L	177.3 (153.7-212.0)	184.0 (153.1-231.4)	175.4 (153.0-207.0)	174.9 (154.9-202.0) †‡
Peripheral arterial oxygen Saturation, %	98.0 (96.0-99.0)	97.0 (96.0-99.0)	98.0 (97.0-99.0) †	98.0 (97.0-99.0) †‡
Respiratory rate,/min	16.0 (16.0-20.0)	17.0 (16.0-20.0)	16.0 (16.0-20.0)	16.0 (16.0-20.0) †‡
Heart rate,/min	81.0 (70.0-94.0)	84.0 (72.0-97.0)	81.0 (70.0-93.0)	80.0 (70.0-92.0) †‡
Systolic blood pressure, mm Hg	140.0 (125.0-158.0)	135.0 (119.8-153.0)	142.0 (128.0-160.0)	141.0 (128.0-160.0) †‡
GCS	15.0 (15.0-15.0)	15.0 (15.0-15.0)	15.0 (15.0-15.0)	15.0 (15.0-15.0) †‡

Values are presented as medians (interquartile ranges) or n (%).

For the P value, † indicates a *p* value < 0.05 versus Tertile 1, ‡ indicates a p value < 0.05 versus Tertile 2. GCS, Glasgow Coma Scale.

**Table 2:** Baseline demographic and clinical characteristics of readmitted patients.

	All (n = 881)	Tertile 1 (n = 293)	Tertile 2 (n = 295)	Tertile 3 (n = 293)
Albumin, g/L	40.5 (35.6-43.7)	<37.6	37.6-42.6	>42.6
Age, year	67 (53-79)	73 (62-82)	69 (54-78) †	59 (41-72) †‡
Sex				
Male, n (%)	450 (51.1)	157 (17.8)	154 (17.5)	139 (15.8)
female, n (%)	431 (48.9)	136 (15.4)	141 (16.0)	154 (17.5)
Triage				
Green, n (%)	256 (29.1)	78 (8.9)	79 (9.0)	99 (11.2)
Yellow, n (%)	357 (40.5)	124 (14.1)	116 (13.2)	117 (13.3)
Orange, n (%)	226 (25.7)	74 (8.4)	88 (10.0)	64 (7.3)
Red, n (%)	42 (4.8)	17 (1.9)	12 (1.4)	13 (1.5)
C-reactive protein, mg/L	1.2 (0.3-5.7)	5.4 (1.3-13.8)	1.0 (0.3-3.4) †	0.4 (0.1-1.2) †‡
Potassium, mmol/L	4.1 (3.8-4.4)	4.0 (3.7-4.5)	4.1 (3.8-4.4)	4.1 (3.8-4.3)
Sodium, mmol/L	137.0 (134.0-139.4)	135.9 (132.0-138.6)	136.9 (134.4-139.0) †	138.1 (135.9-139.8) †‡
Hemoglobin, mmol/L	8.1 (7.1-8.9)	7.0 (6.2-7.9)	8.2 (7.3-8.8) †	8.9 (8.2-9.5) †‡
Creatinine, μmol/L	73.0 (59.0-94.7)	75.0 (58.0-108.4)	75.0 (61.9-96.5)	70.1 (57.1-82.5) †‡
Blood leucocyte count, 109/L	8.9 (7.0-11.9)	9.5 (7.3-13.2)	8.5 (6.7-11.2) †	8.6 (7.0-11.3) †
Lactate dehydrogenase, U/L	183.0 (154.9-228.4)	193.0 (158.8-254.5)	181.7 (153.0-221.5) †	176.9 (154.2-210.9) †
Peripheral arterial oxygen saturation, %	98.0 (96.0-99.0)	97.0 (95.0-98.0)	98.0 (96.0-99.0) †	98.0 (96.0-99.0) †
Respiratory rate,/min	16.0 (16.0-20.0)	18.0 (16.0-20.0)	16.0 (16.0-20.0)	16.0 (16.0-20.0) †
Heart rate,/min	83.0 (72.0-97.0)	85.0 (73.0-100.0)	80.0 (69.0-96.0) †	84.0 (73.0-96.5)
Systolic blood pressure, mm Hg	134.0 (120.0-154.0)	127.0 (113.0-145.0)	137.0 (121.0-158.0) †	138.0 (124.0-158.0) †
GCS	15.0 (15.0-15.0)	15.0 (15.0-15.0)	15.0 (15.0-15.0)	15.0 (15.0-15.0) †

The values are presented as medians (interquartile ranges) or n (%).

For the P value, † indicates a *p* value < 0.05 versus Tertile 1, ‡ indicates a p value < 0.05 versus Tertile 2. GCS, Glasgow Coma Scale.

### Albumin levels and clinical outcomes for the different admission groups

**Fig 1:**
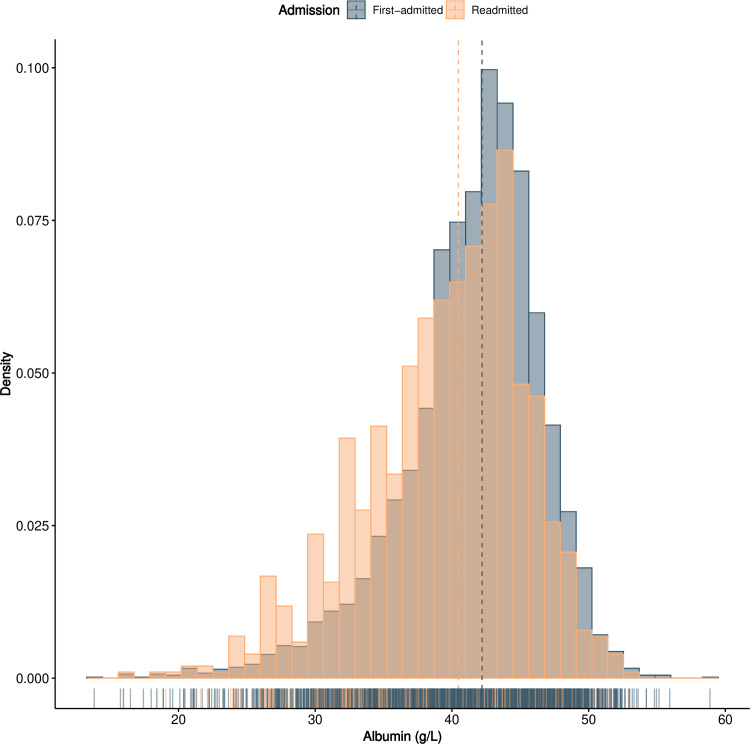
Histogram of albumin in different patients.

As shown in Fig 1, for first-time admitted patients, the median albumin concentration was greater than that for patients who were readmitted (42.20 g/L, IQR 38.91–44.80 g/L vs. 40.47 g/L, IQR 35.62–43.68 g/L, Z = −9.642, *P* < 0.001). The length of hospitalization was used as the follow-up time. The median length of hospitalization was 2 days (range: 1–123 days), and 328 patients died within 30 days. The all-cause mortality rate among patients with unplanned early readmission was greater than that among first-time admitted patients (4.49% vs. 9.86%, $\chi^{2}$ =44.136, *P* < 0.001). In both groups of patients, those who died had low quantile numbers. The mortality rate was higher in the 1st tertile patient group than in the 3rd tertile group (first admission, $\chi^{2}$ =248.542, *P* < 0.001; readmission, $\chi^{2}$ =63.916, *P* < 0.001) (Table 3).

**Table 3:** Proportion of all-cause and death events in patients according to admission and albumin tertiles.

Whole population (N = 6,249)	Tertile 1, Albumin: < 39.8 g/L	Tertile 2, Albumin: 39.8–43.8 g/L	Tertile 3, Albumin: > 43.8 g/L	$\chi^{2}$	*P*
Survival, n (%)	1,834 (87.46)	2,065 (97.77)	2,022 (99.11)	341.286	<0.001*
Death, n (%)	263 (12.54)	47 (2.23)	18 (0.88)		
**First-admission (N = 5,368)**	**Tertile 1, Albumin: < 40.1 g/L**	**Tertile 2, Albumin: 40.1–43.9 g/L**	**Tertile 3, Albumin: > 43.9 g/L**	$\chi^{2}$	***P***
Survival, n (%)	1,605 (89.27)	1,741 (98.08)	1,781 (99.22)	248.542	<0.001*
Death, n (%)	193 (10.73)	34 (1.92)	14 (0.78)		
**Readmitted (N = 881)**	**Tertile 1, Albumin: < 37.6 g/L**	**Tertile 2, Albumin: 37.6–42.6 g/L**	**Tertile 3, Albumin:** **> 42.6 g/L**	$\chi^{2}$	***P***
Survival, n (%)	232 (79.18)	274 (92.88)	288 (98.29)	63.916	<0.001*
Death, n (%)	61 (20.82)	21 (7.12)	5 (1.71)		

The values are presented as n (%).

* *p* value <0.05.

### Role of albumin in the prediction of short-term outcomes in the two admission groups

**Fig 2:**
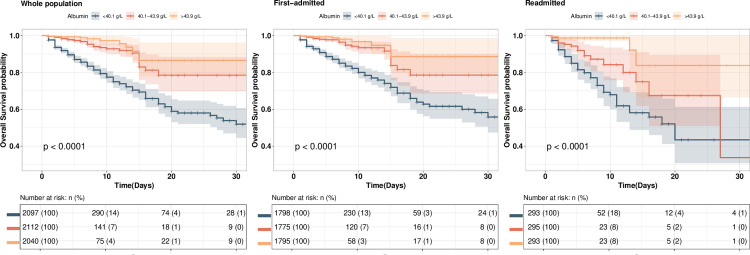
Kaplan-Meier curves for all-cause mortality according to albumin tertiles.

As shown in Fig 2, Kaplan‒Meier analysis revealed that albumin was associated with 30-day mortality in all patients, in first-time admitted patients and in patients with unplanned early readmission. The results of the log-rank test revealed that, for all patients, there were significant differences in 30-day mortality between the patient groups in the different albumin tertiles (*P* < 0.001). For first-time admitted patients, 3rd tertile patients had a lower mortality risk than 2nd tertile patients did (*P* < 0.001); for patients with unplanned early readmission, the mortality risk of the 3rd tertile group was only higher than that of the 1st tertile group (*P* < 0.001).

As shown in Table 4, the patient albumin value was analyzed by Cox proportional hazards regression separately as a continuous variable and as a categorical variable. When albumin was analyzed as a continuous variable, the unadjusted model results revealed that the level of serum albumin (continuous, per 1-unit increase) was associated with the 30-day mortality risk in all patients combined (HR 0.89, 95% CI 0.88–0.90, *P* < 0.001), first-time admitted patients, and patients with unplanned early readmission; however, the results of the covariate-adjusted model revealed that albumin was a significant predictor of mortality in all patients combined (HR 0.93, 95% CI 0.91–0.95, *P* < 0.001) and first-time admitted patients (HR 0.92, 95% CI 0.89–0.94, *P* < 0.001) but not in patients with unplanned early readmission (HR 0.97, 95% CI 0.93-1.01, *P*=0.121). The same result was obtained with the Cox regression model employing albumin as a categorical variable. In addition, sensitivity analysis revealed that the regression analysis results were stable (Table 4, S2 Table, S3 Table, S4 Table and S5 Table in S1 File).

**Table 4:** Association between albumin and all-cause mortality risk via Cox regression analysis.

	Nonadjusted model		Adjusted model		Sensitivity analysis (Forward LR)	
Whole population (N = 6,249) ¶	HR (95% CI)	*P*	HR (95% CI)	*P*	HR (95% CI)	*P*
Continuous per 1 unit increase	0.89 (0.88-0.90)	<0.001*	0.93 (0.91-0.95)	<0.001*	0.92 (0.90-0.93)	<0.001*
Tertile 1 (<39.8 g/L)	Reference		Reference		Reference	
Tertile 2 (39.8–43.8 g/L)	0.25 (0.19-0.35)	<0.001*	0.43 (0.31-0.61)	<0.001*	0.42 (0.30-0.59)	<0.001*
Tertile 3 (>43.8 g/L)	0.13 (0.08-0.20)	<0.001*	0.30 (0.18-0.50)	<0.001*	0.28 (0.17-0.47)	<0.001*
**First-admission (N = 5,368, 85.9%)** ^∮^						
Continuous per 1 unit increase	0.89 (0.87-0.90)	<0.001*	0.92 (0.89-0.94)	<0.001*	0.91 (0.90-0.93)	<0.001*
Tertile 1 (<40.1 g/L)	Reference		Reference		Reference	
Tertile 2 (40.1–43.9 g/L)	0.25 (0.17-0.36)	<0.001*	0.44 (0.30-0.66)	<0.001*	0.38 (0.26-0.55)	<0.001*
Tertile 3 (>43.9 g/L)	0.13 (0.07-0.22)	<0.001*	0.30 (0.17-0.55)	<0.001*	0.26 (0.15-0.45)	<0.001*
**Readmitted (881, 14.1%)**⊥						
Continuous per 1 unit increase	0.91 (0.88-0.94)	<0.001*	0.97 (0.93-1.01)	0.121	0.96 (0.92-1.00)	0.049*
Tertile 1 (<37.6 g/L)	Reference		Reference		Reference	
Tertile 2 (37.6–42.6 g/L)	0.50 (0.30-0.83)	0.007*	1.02 (0.49-2.10)	0.969	N/A	N/A
Tertile 3 (> 42.6 g/L)	0.15 (0.06-0.36)	<0.001*	0.52 (0.15-1.85)	0.312	N/A	N/A

^¶^ The multivariate Cox regression analysis was adjusted for age, triage, c-reactive protein, potassium, sodium, hemoglobin, creatinine, blood leucocyte count, lactate dehydrogenase, peripheral arterial oxygen saturation, respiratory rate, heart rate, systolic blood pressure, and GCS score.

^∮^The multivariate Cox regression analysis was adjusted for age, sex, triage, c-reactive protein, sodium, hemoglobin, creatinine, blood leucocyte count, lactate dehydrogenase, peripheral arterial oxygen saturation, respiratory rate, heart rate, systolic blood pressure, and GCS.

⊥ The multivariate Cox regression analysis was adjusted for age, triage, c-reactive protein, potassium, sodium, hemoglobin, creatinine, blood leucocyte count, lactate dehydrogenase, peripheral arterial oxygen saturation, and GCS score.

* *p* value<0.05.

LR, likelihood ratio.

### Restricted cubic spline (RCS) and threshold analysis

**Fig 3:**
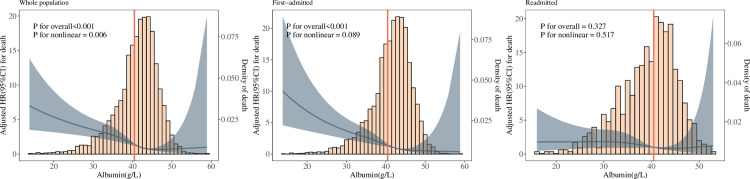
Dose-response relationship between albumin and all-cause mortality by the number of admissions. (For all-cause mortality, covariates including, age, sex, triage, c-reactive protein, pottasium, sodium, haemoglobine, creatinine, blood leucocyte count, lactate dehydrogenasis, peripheral arterial oxygen saturation, respiratory rate, heart rate, systolic blood pressure, and GCS, were adjusted.).

As shown in Fig 3, the relationship between albumin and mortality risk was fitted by an RCS. The results showed that albumin was linearly associated with mortality risk for all patients and first-time admitted patients (*P* < 0.001); the relationship between albumin and mortality risk for all patients could also be fitted with a nonlinear function (*P* = 0.006). However, the relationship between albumin and mortality risk for patients with unplanned early readmission could not be fitted with either a linear (*P* = 0.327) or a nonlinear function (*P* = 0.517). For all patients and first-time admitted patients, the short-term mortality risk increased as the albumin concentration decreased; specifically, when the albumin concentration was less than the median (all patients <42.02 g/L, first-time admitted patients <42.4 g/L), the risk of mortality increased more than when the albumin concentration was greater than the median; however, this trend did not appear in the patients with unplanned early readmission.

## Discussion

Through post hoc analysis of retrospective cohort data, we found that there was a specific association between albumin and the short-term outcomes of ED patients. Albumin affected the short-term mortality risk of patients first admitted to the ED, but the effect on patients with unplanned early readmission was not significant. Among previously published studies, the association between albumin and the short-term outcomes of patients readmitted to the ED is controversial. To our knowledge, this is the first association study between albumin and short-term survival in patients with unplanned early readmission to the ED.

Albumin was negatively associated with the risk of unplanned early readmission in ED patients. A prospective cohort study from Indiana University revealed that for every 10 g/L increase in the serum albumin concentration, the protection against readmission was 0.67 (95% CI, 0.67–0.83) [11]. Another retrospective clinical study from Saudi Arabia revealed that in patients receiving total laryngectomy, a normal preoperative albumin level was associated with a reduced risk of postoperative readmission (OR, 0.51; 95% CI, 0.34–0.67) [31]. The same phenomenon was observed in patients who had undergone lower extremity bypass surgery; patients who had severe hypoalbuminemia (<28 g/L) before surgery had a higher readmission rate than did patients with normal albumin (within 30 days: 40.0% vs. 17.8%, within 90 days: 66.7% vs. 35.6%) [32]. Consistent with previous study results, we found that the albumin level of patients readmitted to the ED was significantly lower than that of first-time admitted patients. Many pathological factors cause a decrease in the level of albumin in patients; these factors can reduce the ability of the liver to synthesize albumin, accelerate the metabolism of serum albumin, and reduce the gastrointestinal absorption of nutrients [14]. However, among the factors that influence the level of serum albumin, inflammation deserves the most attention. Studies on relevant biochemical mechanisms have revealed that the development of inflammation in disease can increase capillary permeability, thereby increasing the penetration of serum albumin into the interstitial space [33,34]. Numerous clinical trials and studies have confirmed that observing and improving the degree of inflammation can reduce the risk of patient readmission [35–37]. Therefore, albumin is often used as a laboratory indicator to assess the risk of readmission in ED patients.

A specific association was detected between albumin and the short-term outcomes of ED patients in this study. Previous studies have revealed an association between albumin and patient survival in many diseases. One population study from Italy revealed that for every 10 g/L decrease in albumin in septic patients in the ED, the 30-day mortality risk increased by 2.991 (95% CI, 1.619–5.525) [11]. In another prospective study of 576 hospitalized patients with heart failure, the 30-day mortality of 160 patients with hypoalbuminemia was 21.8%, which was 2.44 times greater than that of patients with a normal albumin concentration [38]. Interestingly, we found that albumin did not have a significant effect on short-term prognosis among patients who experienced unplanned early readmission to the ED (HR, 0.97; 95% CI, 0.93–1.01; *P* = 0.121), and there was no difference in the risk between the 3rd (> 42.6 g/L) and 1st (<37.6 g/L) albumin tertile groups (HR, 0.52; 95% CI, 0.15–1.85; *P* = 0.312). Although such results have not been reported in previous studies, the conclusions of many studies indirectly support our findings. These studies suggest that the diagnostic performance of albumin for patient survival is limited. First, both effective treatment and condition exacerbation affect the stability of albumin in the short term. Multiple studies have confirmed that even when a patient’s albumin concentration is low at admission, if it returns to normal during subsequent treatment, the patient’s mortality risk is reduced [9,18]. Second, clinical trials have revealed that in both adult and pediatric patients, exogenous albumin infusion has little benefit and might even negatively impact survival in patients with hypoalbuminemia [28,39–43]. In addition, patients with congenital albuminemia typically have serum albumin levels less than 1 g/L; however, this does not affect their lifespan [44]. Moreover, animal experiments have confirmed that analbuminemic rats still have normal reproductive ability [45]. Notably, however, other studies have shown that the prevalence of hypoalbuminemia in patients with disease limits the diagnostic performance of albumin, restricting it to serving only as a surrogate marker for predicting survival [26]. The conclusions of this study suggest that a low albumin level could be used as a warning sign for patients readmitted to the ED, but for ED patients with low albumin levels, it may not be a high-performance indicator of patient survival.

Our study has several limitations. I. the other variables (CRP, Hb and others) and continuous variables’ extreme values might affect the prediction of albumin. II. Only a few covariate indicators that affect the prediction of patient survival were included in our analyses; notably, some imaging indicators with high diagnostic performance were not included. III. The study results were derived from a single-center dataset, and the types of ED patients included were limited; thus, a certain degree of patient selection bias may be present. IV. The study conclusions were derived from the data of Danish patients; the scope of the study must be expanded before extrapolating these findings to patients of other ethnicities. Therefore, high-quality, large-scale, prospective pilot studies are needed to confirm our findings.

## Conclusions

Albumin has predictive value for short-term outcomes in ED patients; however, its utility may be reduced in patients with unplanned early readmission with generally low albumin values. Because the short-term mortality of patients with unplanned early readmission to the ED is greater than that of patients admitted for the first time, these specific differences should not be ignored. Our study results suggest that identifying prognostic biomarkers with high sensitivity and specificity for patients who are readmitted to the ED is clinically important.

## Supporting information

S1 File S1 Table. Results of the normality test. S2 Table. Baseline demographic and clinical characteristics of first admission patients. S3 Table. Baseline demographic and clinical characteristics of readmitted patients. S4 Table. Proportion of all-cause and death events in patients according to admission and albumin tertiles. S5 Table. Association between albumin and all-cause mortality risk via Cox regression analysis.
(ZIP)
