# Supplementary material for: Association between albumin and short-term outcomes of unplanned early readmission emergency department patients: A retrospective cohort study
Source: PLoS One. 2025 Jul 24;20(7):e0327501. doi: 10.1371/journal.pone.0327501 (PMC12289061; doi:10.1371/journal.pone.0327501)
Supplement: S1 File — S1 Table. Results of the normality test. S2 Table. Baseline demographic and clinical characteristics of first admission patients. S3 Table. Baseline demographic and clinical characteristics of readmitted patients. S4 Table. Proportion of all-cause and death events in patients according to admission and albumin tertiles. S5 Table. Association between albumin and all-cause mortality risk via Cox regression analysis. (ZIP) [file pone.0327501.s001.zip › Supporting Information/S4 Table.docx]

**S4 Table. Proportion of all-cause and death events in patients according to admission and albumin tertiles**

|  | **Group 1, Albumin: <35.0 g/L** | **Group 2, Albumin: >=35.0 g/L** | $\boldsymbol{\chi}^{\boldsymbol{2}}$ | **P-value** |
| --- | --- | --- | --- | --- |
| **Whole population (N=6,249)** |  |  |  |  |
| Survival, n (%) | 597 (78.14) | 5,324 (97.06) | 341.286 | <0.001* |
| Death, n (%) | 167 (21.86) | 161 (2.94) |  |  |
| **First admission (N=5,368)** |  |  |  |  |
| Survival, n (%) | 452 (79.72) | 4675 (97.38) | 368.737 | <0.001* |
| Death, n (%) | 115 (20.28) | 126 (2.62) |  |  |
| **Readmitted (N=881)** |  |  |  |  |
| Survival, n (%) | 145 (73.60) | 649 (94.88) | 77.814 | <0.001* |
| Death, n (%) | 52 (26.40) | 35 (5.12) |  |  |

The values are presented as n (%).

* *p value <0.05.*
