# Supplementary material for: Association between albumin and short-term outcomes of unplanned early readmission emergency department patients: A retrospective cohort study
Source: PLoS One. 2025 Jul 24;20(7):e0327501. doi: 10.1371/journal.pone.0327501 (PMC12289061; doi:10.1371/journal.pone.0327501)
Supplement: S1 File — S1 Table. Results of the normality test. S2 Table. Baseline demographic and clinical characteristics of first admission patients. S3 Table. Baseline demographic and clinical characteristics of readmitted patients. S4 Table. Proportion of all-cause and death events in patients according to admission and albumin tertiles. S5 Table. Association between albumin and all-cause mortality risk via Cox regression analysis. (ZIP) [file pone.0327501.s001.zip › Supporting Information/S5 Table.docx]

**S5 Table. Association between albumin and all-cause mortality risk via Cox regression analysis**

|  | **Nonadjusted model** | | **Adjusted model** | | **Statistical analysis (Forward LR)** | |
| --- | --- | --- | --- | --- | --- | --- |
| Whole population (N=6,249) ¶ | HR (95% CI) | *P* | HR (95% CI) | *P* | HR (95% CI) | P-value |
| Tertile 1 (<39.8 g/L) | Reference |  | Reference |  | Reference |  |
| Tertile 2 (39.8-43.8 g/L) | 0.22 (0.18-0.28) | <0.001* | 0.47 (0.36-0.62) | <0.001* | 0.46 (0.35-0.60) | <0.001* |
| First admission (N=5,368, 85.9%) ∮ |  |  |  |  |  |  |
| Group 1 (<35.0 g/L) | Reference |  | Reference |  | Reference |  |
| Group 2 (>=35.0 g/L) | 0.21 (0.16-0.27) | <0.001* | 0.44 (0.32-0.60) | <0.001* | 0.39 (0.29-0.52) | <0.001* |
| Readmitted (881, 14.1%) ⊥ |  |  |  |  |  |  |
| Group 1 (<35.0 g/L) | Reference |  | Reference |  | Reference |  |
| Group 2 (>=35.0 g/L) | 0.31 (0.20-0.48) | <0.001* | 0.70 (0.41-1.20) | 0.191 | N/A | N/A |

¶ The multivariate Cox regression analysis was adjusted for age, triage, c-reactive protein, potassium, sodium, hemoglobin, creatinine, blood leucocyte count, lactate dehydrogenase, peripheral arterial oxygen saturation, respiratory rate, heart rate, systolic blood pressure, and GCS score.

∮ The multivariate Cox regression analysis was adjusted for age, triage, c-reactive protein, potassium, sodium, hemoglobin, creatinine, blood leucocyte count, lactate dehydrogenase, peripheral arterial oxygen saturation, respiratory rate, heart rate, systolic blood pressure, and GCS.

⊥ The multivariate Cox regression analysis was adjusted for age, triage, c-reactive protein, potassium, sodium, hemoglobin, blood leucocyte count, lactate dehydrogenase, peripheral arterial oxygen saturation, and GCS.

* *p value <0.05.*
